# Enteric microbiome and obesity: a multidimensional narrative review

**DOI:** 10.1099/mgen.0.001562

**Published:** 2025-11-27

**Authors:** Bidisha Barat, Kinanka Ghosh, Bipin Kulkarni, Prabir Banerjee, Kanjaksha Ghosh

**Affiliations:** 1 The University of Chicago, Chicago, IL, 60637, USA; 2 Groupon, Inc., Chicago, IL, 60601, USA; 3 National Institute of Immunohematology, KEM Hospital, Mumbai, Maharashtra, 400012, India; 4 Consultant Endocrinologist Birmingham NHS Trust Hospitals, Birmingham, UK

**Keywords:** appetite, chronic inflammation, enteric microbiome, epigenetics, epigenetic changes, food-seeking behaviour, hunger, immunomodulation, modulation, multisystem effect, neurotransmitters, obesity, pathologic condition, satiety

## Abstract

Obesity (henceforth called the pathologic condition) is a global epidemic affecting nearly 20% of adults worldwide and transcends genetic, ethnic and civilizational barriers in this era of globalization. Various ways of stemming the progress of the disease have been considered. One significant finding in this condition is an altered enteric microbiome. This review elucidates multiple mechanisms by which an altered enteric microbiome may contribute to the pathologic condition. Key roles include the microbiome’s ability to process dietary roughage into absorbable nutrients, modulate intestinal physiology, enhance nutrient absorption and influence the endocrine function of adipose tissue and the liver to regulate appetite and hunger. The gut microbiome also interacts with the entero-insular axis, optimizing food utilization, and communicates with the central nervous system to alter appetite, satiety and food-seeking behaviour. Additional mechanisms include immunomodulation, chronic inflammation, epigenetic regulation and the production of vitamins and antioxidants. Promising therapeutic avenues, including engineering beneficial gut bacteria, developing targeted probiotic formulations and designing specialized food programmes can help combat this pathologic condition and potentially provide effective, non-invasive strategies to address this widespread condition.

**Impact Statement**

In this manuscript, we address critical gaps in understanding the enteric microbiome’s pivotal role in the pathophysiology of obesity which has become a global health crisis. By integrating evidence from microbiome composition, metabolic functions, immune modulation and epigenetic factors, we demonstrate the gut microbiome’s role as a dynamic neuroendocrine and metabolic organ. The review highlights novel therapeutic avenues, including the engineering of beneficial gut bacteria, targeted probiotic formulations and dietary interventions, as promising non-invasive strategies to combat obesity. Our work aims to inform future research and clinical applications, advancing the field of microbial genomics by linking microbiota-driven mechanisms with systemic metabolic outcomes and fostering innovative approaches to address obesity with precision and sustainability.

## Data Summary

The authors confirm that all supporting data have been provided within the article. All the data is available in the cited literature.

## Introduction

The developed world is currently experiencing a widespread epidemic of obesity. Irrespective of the regions studied, the prevalence of people with the pathologic condition was found to be increasing over time everywhere in the world except in the Western Pacific Island [1]. Presently, there are 1 billion people with this pathologic condition in the world, now defined as a chronic, systemic disease characterized by excess adiposity, not merely reflected by body mass index (BMI) alone, with clinical obesity requiring evidence of adipose-mediated tissue or organ dysfunction. Diagnosis should involve body fat assessment rather than BMI alone when feasible; a BMI ≥30 kg m² still serves as a practical screening threshold in most settings, especially for severe adiposity cases (>40 kg m²) where adiposity can be presumed without further testing [2]. The pathologic condition is increased across the spectrum of age and sex. It is believed that there could be genetic, endocrine, metabolic and other disease-associated causes for this pathology in individual cases. The present epidemic of the pathologic condition has been linked to a sedentary lifestyle, abnormal dietary composition and food habits in the background of the genetic and endocrine landscape of the individual [1]. However, those who have attempted to treat the common form of this condition using drugs, diet and lifestyle modifications often find that their efforts fail over time. Barriers to manage this pathologic condition have been described elsewhere [3]. Bariatric surgery is one of the few procedures shown to sustain weight loss over a longer period [4]. Standard knowledge of the pathophysiology of the condition and its current management has been discussed in several current reviews [5,6]. Recently, several drugs have been developed targeting GLP-1 receptor agonist, semaglutide [7] or both GLP-1 and GIP agonists, tirzepatide [8], in 1 weekly dose to reduce body weight over a sustained period. Retatrutide, which also targets glucagon receptor (GCGR) in addition to GLP-1 and GIP receptors, produced yet a higher degree (24%) of sustained weight loss [9]. All these medicines should be combined with dietary guidance and exercise. Before the advent of current insulin mimetics and incretin mimetics, the drugs commonly used to treat this pathological condition included sympathomimetics, MAO inhibitor-like compounds, cannabinoid receptor agonist (such as rimonabant), antiepileptic compounds (like topiramate) or drugs that interfere with lipolysis in the intestine interfering in its absorption (such as orlistat) [10]. None of these medications were very effective and had significant side effects. Rimonabant was withdrawn due to increased risk of depression and suicidality [11]; topiramate can cause cognitive impairment and paresthesia [12], and orlistat is associated with gastrointestinal side effects**,** including oily stools and faecal urgency [13]. The weight loss with these medicines was initially 5–8% and was rarely sustained; however, even modest reductions of this magnitude have been associated with clinically meaningful improvements in glycaemic control, lipid profiles and blood pressure, despite not achieving a normal BMI [14].

The most commonly encountered form of this pathological condition is primarily caused by an imbalance between energy intake, derived from absorbed food, and energy expenditure through heat generation, bodily functions and physical activity. Therefore, the management approach for individuals with obesity, in the absence of evident endocrine, metabolic or genetic causes, focuses on addressing the mismatch between food consumption and energy expenditure. Management of the intake in the form of dietary management and output in the form of added work/exercise should have taken care of pathologic condition. However, this was not the case; it was found that individuals with this condition who initially lost some weight gained it back despite continued management of the same. Studies show that in the course of time, the body makes metabolic adjustments to suit the intake [15] through adaptive thermogenesis. Moreover, it was assumed that the fibre (roughage) in the food cannot contribute to the energy input in the host in any meaningful way.

The last three decades of research show that this may not be true and enteric microbiome/microbiota may have a significant role in the pathogenesis of the condition and understanding this well may offer newer ways of controlling body mass in addition to what has already been achieved. The present review discusses various ways the enteric microbiome may contribute to the current pathological epidemic. In this article, we will be using the term microbiota and microbiome interchangeably, though microbiota means the sum total of culturable organisms and microbiome means the totality of DNA extractable from the faecal material of the patient. In that sense, the microbiome represents a more extensive span of organisms than the microbiota. The review was created by conducting a search through PubMed since 1990 using keywords such as enteric microbiome, culturomics, obesity, twin studies, community studies, molecular techniques, metagenomic and molecular techniques.

## Enteric microbiome

The enteric microbiome is seeded from the mother’s gastrointestinal tract and genital tract at the time of vaginal delivery, including *Bacteroides*, *Bifidobacteria* and *Lactobacilli*, and from the skin flora during a caesarean operation, such as *Staphylococcus* and *Corynebacterium* species. Microbiota in the breast milk and its influence on the microbiome also modulates its components with the growth of child. Strategies to restore a typical microbial profile in C-section–delivered infants include exclusive breastfeeding, which promotes *Bifidobacteria* and *Lactobacilli* via human milk oligosaccharides [16]. Vaginal microbial transfer has shown potential to partially restore maternal-like microbiota [17], though long-term safety is still under review. Recently, maternal faecal microbiota transplantation (FMT) has been shown to rapidly normalize gut microbiota in caesarean-born infants, including restoration of *Bacteroides* colonization [18]. As a child grows, their microbiome transitions from being dominated by *Bifidobacteria* and *Lactobacillus* during the first 3–4 years to a more adult-like configuration represented by six phyla: *Bacillota* (formerly *Firmicutes*), *Bacteroidota* (formerly *Bacteroidetes*), *Actinomycetota* (formerly *Actinobacteria*), *Pseudomonadota* (formerly *Proteobacteria*), *Fusobacteriota* (formerly *Fusobacteria*) and *Verrucomicrobiota*. Among these, *Bacillota* and *Bacteroidota* are the most prevalent [19]. Additionally, the human gut microbiota includes fungi, viruses, phages and archaea, particularly *Methanobrevibacter smithii* [20,22]. The transition to an adult-like microbiome is influenced by weaning, dietary diversification that promotes the dominance of microbial species capable of metabolizing complex carbohydrates, proteins and fibres in place of milk-adapted species such as *Bifidobacteria*, environmental exposures (e.g. pets and siblings), early antibiotic use and immune maturation [23]. Host genetics, such as FUT2 secretor status, and geographic dietary patterns influence mucin glycosylation, shaping gut microbial colonization [24].

These six phyla of microbes form the core microbiome and remain reasonably present in all the population groups. Some studies suggested that ratios of *Prevotella* to *Bacteroides* fairly represent the diversity of the microbiome from one host to another, but it is not universally agreed upon. The above-mentioned and some other studies suggest the enteric microbiome changes with age, in response to different foods, when challenged with various antibiotics and in response to many environmental changes. It differs from one member to another member of the family as well as in populations studied in various locations of the world [25,26].

Some contribution from host genes in structuring the details of the enteric microbiome undoubtedly exists [27,28]. In cross-faecal transplantation experiments in mice, it has been shown that a lean phenotype can be produced in mice with the pathologic phenotype, following faecal transplantation from a lean phenotype animal. The present epidemic of obesity across the world demonstrates [1,2] the community effect of the microbiome. This has been described in some studies, meaning that being in a family or community leads to the acquisition of a similar enteric microbiome in many members of the family [21,22, 29,32]. The predominant microbial community in the microbiome across the world has been well described elsewhere [29].

The study of the enteric microbiome and its functional interaction is hampered by the limitations of culture techniques. Scientists use data from 16S ribosome typing to understand the general phylotype of the microbiome. At present, it is understood that more than 1,000 phylotypes may represent the enteric microbiome with fewer than a few hundred culturable ones. To improve this situation, a subject of microbiome culturomics is developing [33,37] with the introduction of newer culture media like YCFA media (yeast, casitone and fatty acids supplemented by carbohydrates from potato or other sources); similarly, media enriched by chicken blood and filtered rumen fluid have also been used. Lagier *et al*. [33] used various modifications of newer media but could culture only half of the phyla of microbes represented in the enteric microbiota. The genomic load from this gut microbiota is 100 times more than the human genome itself.

What is the impact of this 100-fold greater microbial genomic load in the intestine? It seems that these microbes together through their complementary, synergistic and at times competitively antagonistic biochemical machinery and modifying epigenetic and transcriptional activities work as additional endocrine, paracrine, energy-producing, vitamin-contributing and immunologically modulating organ. The enteric microbiome is a neuroendocrine coordination organ that is outside our body but influences innumerable bodily functions. For a detailed discussion of these functions, several current reviews are available [38,39]. DNA sequencing alone cannot provide a complete understanding of previously unculturable organisms, including their diversity, transcriptomic and metabolomic profiles. Newer culture techniques are unravelling organisms in their totality of structure and function. Non-culture techniques like massively parallel genome sequencing coupled with strong statistical programmes and artificial intelligence applications are illuminating the yet unexplored areas of microbiota [40], supplemented by more refined techniques of molecular discovery like mass spectroscopy which is contributing to understanding the metabolome of the microbiota.

All these techniques and the creation of biochemical profiles from the complementarity of products identify a large number of enzymes capable of breaking undigested fibres in the food, complex sugars, mucin, lipids, etc. Biotransformation of various chemicals ingested with food or drugs, bile salt metabolism and generation of small chemicals produced through bacterial metabolism act as ligands to distant targets in the human host. These materials additionally stimulate a network of nerve endings in the gastrointestinal tract and in distant sites, including the brain.

Some of the chemicals produced can also act as epigenetic modifiers. Metabolic modulation by some of these chemicals through receptors for aryl hydrocarbons and by conduction of oxidation-reduction reactions through the production of hydrogen, methane, hydrogen sulphide and nitric oxide adds another dimension to the physiology of the gut and may produce various systemic effects. A variety of endotoxins produced through the secretion and death of these organisms work in tandem to create a network of influence on the host.

## Small intestinal microbiome and obesity

While most microbiome studies have traditionally focused on the colon due to ease of access via faecal sampling, emerging evidence underscores the critical importance of the small intestinal microbiome (SIM) in host metabolism and the pathogenesis of obesity. The SIM, primarily residing in the duodenum and jejunum, is characterized by lower microbial biomass but higher metabolic activity due to rapid nutrient turnover and its close proximity to absorptive epithelial surfaces. Unlike colonic communities, SIM taxa are enriched in facultative anaerobes and exhibit functional profiles tightly linked to nutrient sensing, bile acid metabolism and hormonal regulation.

Recent studies using direct mucosal biopsies, capsule endoscopy and duodenojejunal fluid sampling have demonstrated that SIM composition correlates more strongly with dietary patterns, glycaemic control and adiposity than faecal microbiota [41]. In a mechanistic breakthrough, Wang *et al*. showed that small intestinal microbes reprogramme host lipid metabolism by downregulating the long noncoding RNA *Snhg9* in enterocytes, which in turn enhances PPAR*γ* activity and promotes lipid absorption and fat accumulation [42]. A comparative metagenomic and metabolomic analysis revealed that the duodenojejunal microbiome harbours microbial pathways and metabolic signatures more tightly associated with host metabolic phenotypes than either oral or faecal microbiota [43].

Collectively, these findings underscore the SIM’s role as a dynamic metabolic interface that mediates bile acid deconjugation, modulates incretin hormone secretion (e.g. GLP-1 and GIP) and regulates nutrient uptake and energy harvest. As such, the SIM presents a compelling target for microbiome-based diagnostics and therapeutic interventions aimed at preventing or reversing obesity.

## Gut bacteriophages and microbiome modulation

In addition to bacteria and archaea, the enteric microbiome harbours a vast array of bacteriophages, viruses that infect and regulate bacterial populations. Recent clinical studies have demonstrated that individuals with obesity or type 2 diabetes exhibit a significant reduction in gut virome richness and diversity, particularly in dsDNA phages belonging to the Caudovirales order [44]. Yang *et al*. also observed that the ecological interactions between bacteriophages and their bacterial hosts are weakened in these individuals, suggesting impaired top-down viral regulation of the microbiome. This loss of virome-bacteriome network stability may contribute to reduced bacterial diversity and metabolic imbalance. Although mechanistic details remain to be elucidated, lysogenic phages are hypothesized to influence host immune signalling and intestinal barrier function [44]. Looking forward, engineered phage-based therapies could offer a novel strategy to selectively target pathobionts, restore microbial balance and modulate metabolic outputs in obesity.

## Obesity and enteric microbiome

Obesity is the final long-term result of the net excess of energy input as food and energy output by the body in terms of basal metabolism including thermogenesis and external work. The net excess energy from food accumulates in the body and is stored as glycogen in the muscle and liver. The amount of glycogen that can be stored is limited, but a large amount of this excess energy can be stored in different compartments of adipose tissues. Different areas and compartments of adipose tissue store and mobilize fats differently under different neurohormonal stimulation. Examples of such different stores are visceral adipose tissue, brown adipose tissue and adipose tissue under the skin and around the musculoskeletal system. Proteins normally do not accumulate in the body except during the growing phase of the host, and after this phase is over, protein is used mostly to replace the day-to-day wear and tear of various tissues.

**Fig. 1.**
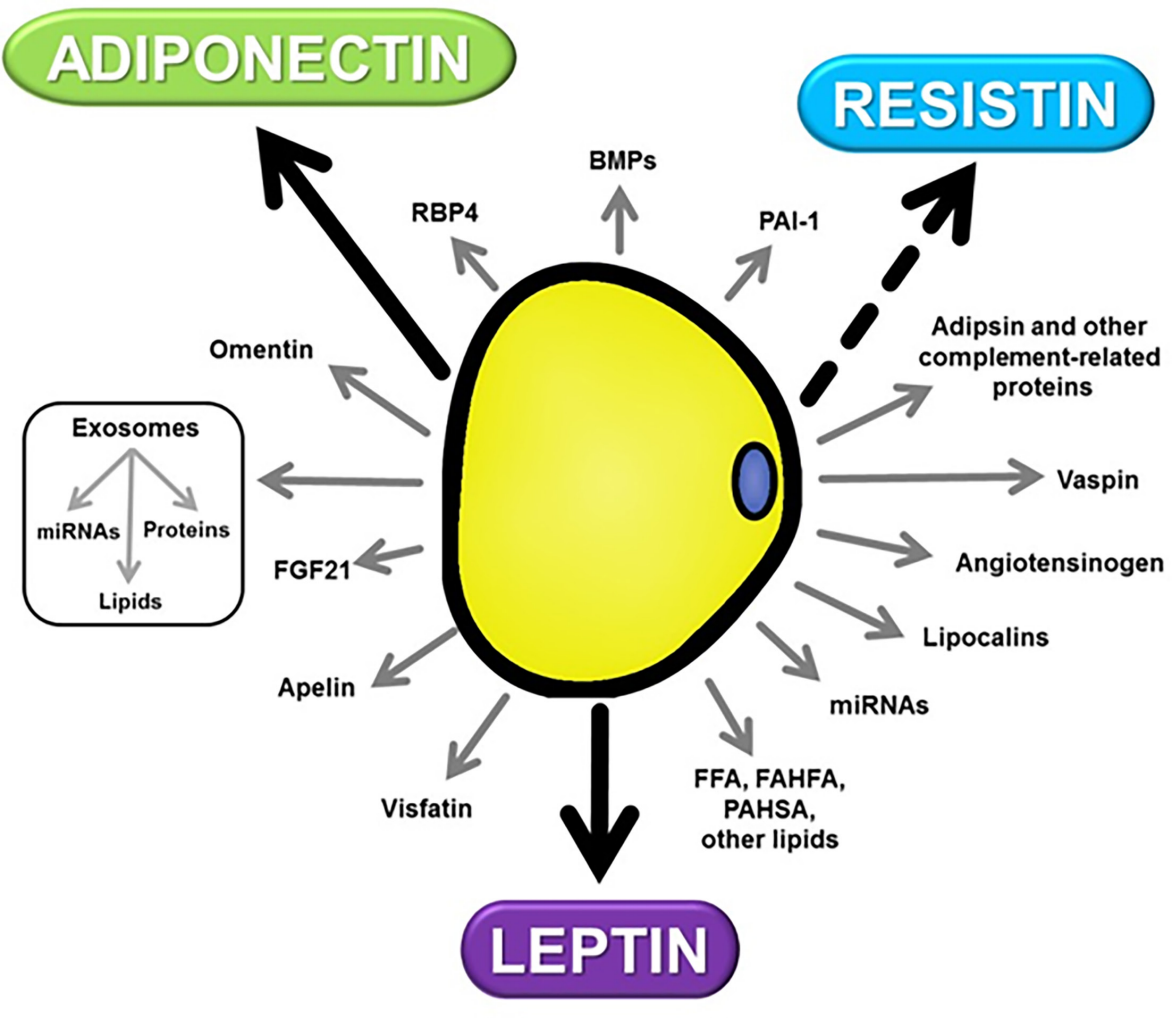
The adipocyte secretome. Fat cells express and release numerous proteins, lipids and nucleic acid factors that can act on other nearby or distant tissues within the body in a paracrine or endocrine manner. Leptin, adiponectin and resistin are highlighted here because they are exclusively secreted from mouse adipocytes, while the other factors can also be secreted from other cell types. The arrow-headed line representing secretion of resistin is dashed since in humans, macrophages, and not adipocytes, primarily produce this adipokine.

Adipose cells accumulated during foetal and childhood periods have the capability of remaining dormant or can accumulate excess fat, which is associated with microbiome alterations termed dysbiosis, contributing to adipose tissue inflammation and metabolic dysfunction. Fat cells either can increase in size (hypertrophy) or may increase in numbers (hyperplasia) from resident adipose stem cells. Major adipose tissue in the body that contributes to this phenomenon is called white adipose tissue; others, i.e. brown, beige and pink adipose tissues, do not contribute to the pathologic condition but are mainly involved in thermogenesis and milk secretion (pink adipocytes) [45]. Adipocytes in the body are one of the largest endocrine organs and act in concert with the liver, intestine, muscle, pancreas and brain to control hunger and appetite, fat deposition and metabolism as well as modulate the intestinal absorption capabilities [46,49] and immunomodulation. In all these actions, the enterobiome significantly contributes almost like another interacting external organ of the host. This will be apparent in the discussion that follows under respective headlines, all of which in some way contribute to the pathologic condition under discussion. Innumerable neurohumoral material that is elaborated from adipose tissue is shown in Fig. 1.

### Ability of the gut microbiome to process roughage into food

The gut microbiome can process the unabsorbed material that passes into the colon, which may consist of soluble fibres, complex carbohydrates, proteins and complex lipids such as phospholipids, sphingolipids and glycolipids. In addition, the microbial metabolic products like amino acids, short-chain fatty acids (SCFAs), hydroxy fatty acids, short absorbable peptides, polyamines, monoamines, vitamins and aryl compounds as well as other xenobiotics through biotransformation and diet-derived compounds like polyphenols that undergo microbial transformation are available either as nutrient source or as ligands for various biological functions to the host. While the gut microbiota does not synthesize polyphenols, it converts dietary polyphenols into bioactive metabolites such as urolithins and phenolic acids, which modulate inflammation, energy metabolism and gene expression [50].

Soluble fibres in the roughage contain xylans, mannans, arabinans, xyloglucan, galactomannan and fructo-oligosaccharides. They can be broken down by various members of the microbiome [51]. Lactobacilli produce lactases, proteases, peptidases, fructanases, amylases, bile salt hydrolases, phytases and esterases [52]. Cellulose, also a very important component of the diet and roughage, can be broken down to simple sugars and fatty acids by the cooperative action of the gut microbiome where *Bacteroides* such as *Bacteroides thetaiotaomicron* and *Bacteroides ovatus* through their interacting polysaccharide-digesting enzymes can process cellulose through hemicellulose and pectins in diet to simple carbohydrates. *Prevotella copri*, *Collinsella aerofaciens* and *Blautia wexlerae* also take part in this digestive process [53,56]. Fatty acids produced can be converted into fat in adipose tissue and the liver. In the human intestine, this conversion may not be happening to a great extent, but given the availability of those complementary bacteria in the gut microbiome, cellulose digestion in the colon is a distinct possibility.

Mucus is an important product of the intestine, and its major component, mucin, is a complex mucopolysaccharide having O-linked and N-linked sugar branches along with many other components including proteins. There are many classes of mucins, but broadly one type sticks to the cell membranes and protects intestinal cells from bacterial invasion and also acts as an immune barrier, and the other variety called MUC2 facilitates the passage of stool in the colon by binding with water and making the faeces a slimy material easier to expel [57].

While the majority of dietary protein is digested and absorbed in the small intestine, a variable fraction ranging from ~6 to 18 g per day can escape digestion and reach the colon depending on factors such as dietary composition, food processing and individual digestive efficiency [58]. This residual protein includes both dietary proteins and endogenous components such as mucin, and this can be digested by several members of the microbiome such as *Akkermansia muciniphila*, producing amino acids, peptides and simple carbohydrates [59]. While canonical amino acid biosynthesis in gut microbes relies on central carbon metabolism such as glycolysis and the tricarboxylic acid cycle, some studies have proposed that cross-fed intermediates derived from fatty acid fermentation may contribute indirectly to amino acid pools, though this remains a speculative area requiring further validation [60]. In addition, the dying bacterial cell, as well as intestinal epithelial cells, also contributes to the amino acid and peptide pools to the microbiome. Colonic mucosa is not very efficient in absorbing amino acids through various amino acid transporters, but a small amount of amino acid is absorbed through this route. Peptides are absorbed better. Similarly, the microbiome is also involved in fat synthesis and storage by the host [47,49,61, 62].

### Changing the physiology of the intestinal tract

The enteric microbiome through its various products can change the motility of the intestine and the tone of various sphincters, slowing or speeding up the transit of food [63,64]. Dysbiosis associated with an altered enteric microbiome has been described in intestinal motility disorders like irritable bowel syndrome. Slowing down intestinal motility may be associated with more complete absorption and digestion of food. Moreover, the altered motility by itself has been shown to change the structure of the microbiome itself [65]. Studies have shown that individuals with slowed intestinal transit exhibit elevated levels of methanogenic archaea, particularly *M. smithii*. Methane production by these microbes has been linked to delayed gastrointestinal motility, which may prolong nutrient absorption time and facilitate greater energy extraction. This slowed transit can also promote the overgrowth of bacterial populations involved in fermentation and absorption, potentially contributing to abnormal microbial colonization. Notably, increased abundance of methanogenic archaea is one of the distinguishing features observed in the gut microbiota of obese individuals compared to lean controls [66].

Enterocyte physiology, particularly its generation and differentiation into different functional cells, specifically for lipid absorption, amino acid absorption, production of enteroendocrine L cells and enterochromaffin cells and mucus-producing cells is important. The life span of these cells can all be modulated by the enteric microbiome through the production of targeted molecules and elaborating the intestinal cell protection type of mucus [67]. Microbiome acting through the intestinal immune system via modulation of its vast cytokine network also controls the differentiation of enteric stem cells into different functional units and by doing that controls a vast amount of functions of the intestine [68]. Many dimensions of gut physiology have been shaped by the enteric microbiome through modulation of the nervous system and glial cells of the gastrointestinal tract [69]. The vagus nerve, a master nerve supplying a large part of the gastrointestinal tract, originates from the brain and integrates various impulses to control gastrointestinal function. Enteric microbiome from the intestine can also influence various gastrointestinal functions via the vagus nerve through the brain [70].

### Improving food absorption and influencing the enteroinsular axis to improve food utilization

The enteric microbiome uses multiple mechanisms to improve the absorption of digested food [49]. It uses several mechanisms such as slowing the intestinal transit time, improving the bile salt output and secretion, elaborating novel biosynthetic pathways [71], changing the permeability of the intestine [72] and improving the enterocyte biology [67,68]. All the above-mentioned functions can contribute to this pathologic condition. In the same way, the microbiome can be altered therapeutically to antagonize some such functions to counter the pathologic condition.

**Fig. 2.**
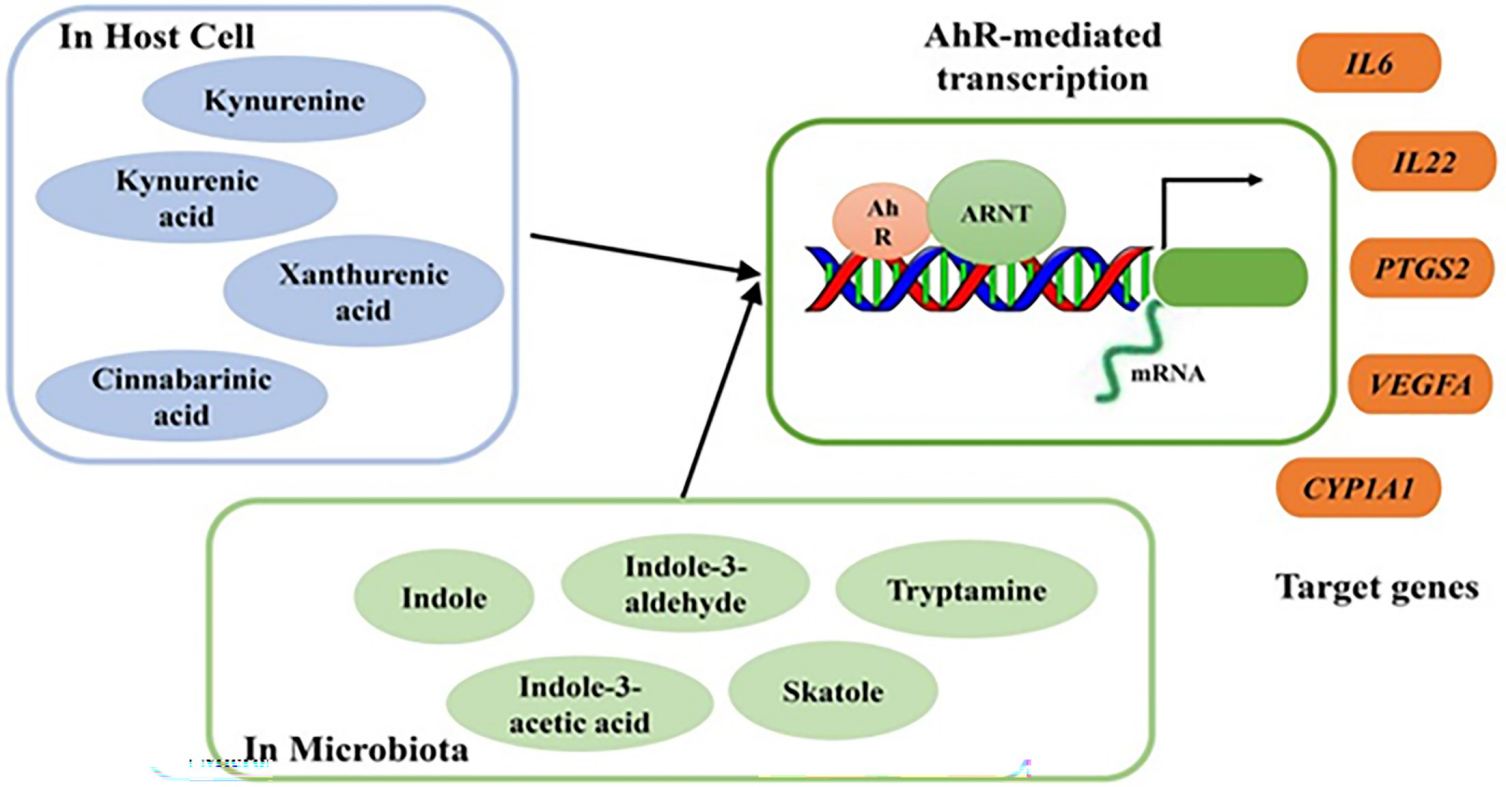
Overview of endogenous and microbial tryptophan metabolites serving as ligands for the aryl hydrocarbon receptor (AhR). This interaction leads to nuclear translocation of AhR and subsequent activation, triggering the transcription of target genes such as IL-6, IL-22, prostaglandin G/H synthase 2 (PTGS2), vascular endothelial growth factor A (VEGFA) and cytochrome P450 1A1 (CYP1A1). Reproduced from Figure 2 in [83]. CC BY (https://creativecommons.org/licenses/by/4.0/).

Enteric microbiome influences the increased production of incretins through the action of their SCFAs on free fatty acid receptors 2 and 3 and GPR43 on L cells elaborating glucagon-like peptides GLP-1 and some peptides like PYY, improving insulin secretion and glucose homeostasis [73,74]. These microbiome-derived SCFAs also stimulate production of ghrelin, which is primarily secreted by the stomach and plays a key role in increasing appetite and inhibiting production of leptin, which produces satiety and termination of food intake. Some other ligands like imidazole propionic acid produced (a product from amino acid histidine) by enteric bacteria can inhibit insulin signalling via mTORC1 [75], contributing to insulin resistance and the pathologic condition (Fig. 2).

### Modulating endocrine function of adipose tissue and liver improving appetite and hunger

The gut microbiome using several mechanisms can influence appetite/hunger/satiety such as altering the ability of the adipose tissue and liver to produce various hormones, i.e. adipokinin, leptin and adiponectin. It also stimulates fat and glycogen deposition in the liver [47,76,79], and some of these enterobiome-related metabolites predispose to non-alcoholic fatty liver disease, a condition which is often associated with pathologic condition [79].

Fat storage in adipose and in various tissues is also in part controlled by the composition of enteric microbiome [77]. The complexity of adipocyte secretome which not only works through the liver but also through many other tissues is influenced by the enteric microbiome products and depending on the situation can contribute to the pathologic or lean phenotype [80,81]. This is well represented in Figs1  3. This is partly influenced by microbial metabolic products and their influence on the liver as well as through immune and non-immune cells including adipocytes.

**Fig. 3.**
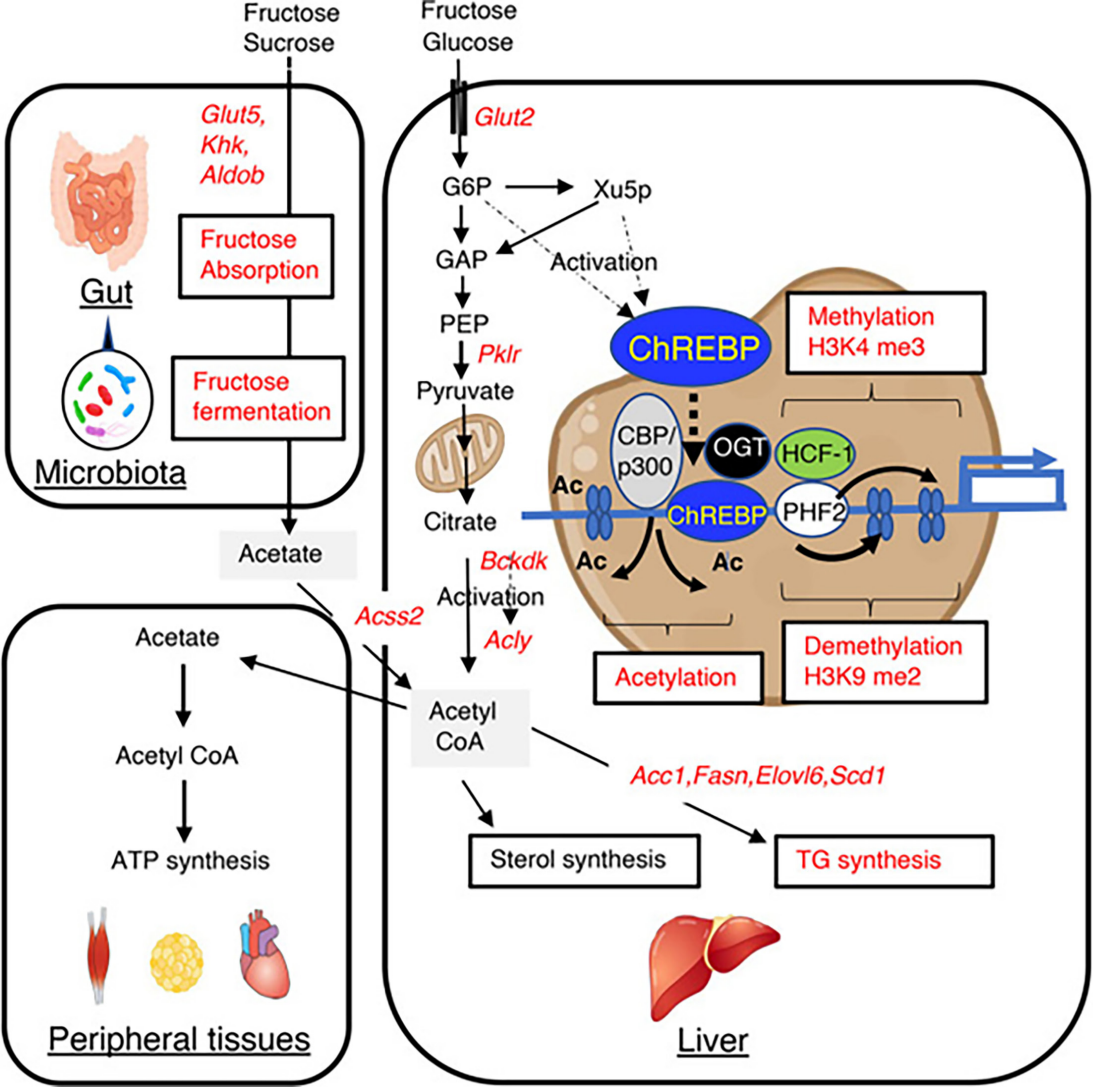
Dietary impact on lipogenesis and gut microbiota interaction. Feeding on a high-fructose/high-sucrose diet triggers an increase in *de novo* lipogenesis by influencing the expression of lipogenic genes and utilizing acetate derived from the gut microbiota. Under normal circumstances, fructose is absorbed and converted into glucose and lactate. The portal vein receives glucose, lactate and a small portion of fructose. Once in the liver, glucose and fructose activate the transcriptional activity of ChREBP by elevating the concentrations of glucose and fructose-derived metabolites like xylulose-5-phosphate and glucose-6-phosphate. This activation leads to the upregulation of lipogenic genes such as Acc1, Fasn, Elovl6 and Scd1. Consequently, this increased expression drives the conversion of glucose and fructose into acetyl CoA and fatty acyl CoA within the liver. Fructose that is not absorbed in the small intestine enters the colon and subsequently the portal vein. In the liver, acetate is transformed into acetyl CoA by acyl-coenzyme A synthetase short-chain family member 2 (encoded by Acss2). This acetyl CoA is then utilized for the synthesis of fatty acyl CoA, sterols and histone acetylation processes. Furthermore, ChREBP transcriptional activity is subject to regulation by acetyl CoA and uridine diphosphate-*N*-acetylglucosamine, through acetylation and O-GlcNAcylation, respectively. These components play a pivotal role in epigenetic regulation, impacting processes like histone acetylation and histone methylation. Key molecular entities involved in this intricate process include GLUT5 (glucose transporter 5), KHK (ketohexokinase), ALDOB (aldolase B), GLUT2 (glucose transporter 2), PKLR (liver-type pyruvate kinase), ACSS2 (acyl-coenzyme A synthetase short-chain family member 2), G6P (glucose 6-phosphate), Xu5P (xylulose 5-phosphate), ACLY (ATP citrate lyase), ACC1 (acetyl CoA carboxylase), FASN (fatty acid synthase), ELOVL6 (fatty acid elongase 6), SCD1 (stearyl CoA desaturase), BCKDK (branched-chain ketoacid dehydrogenase kinase), CBP (CREB binding protein), OGT [O-linked *N*-acetylglucosamine (GlcNAc) transferase], HCF-1 (host cell factor-1), PHF2 (plant homeodomain finger 2), H3K4me3 (trimethylated H3K4) and H3K9me2 (dimethylated H3K9). Reproduced from Figure 1 in [139]. CC BY (https://creativecommons.org/licenses/by/4.0/).

### Acting on the central nervous system to alter appetite/hunger and satiety and food-seeking behaviour

**Fig. 4.**
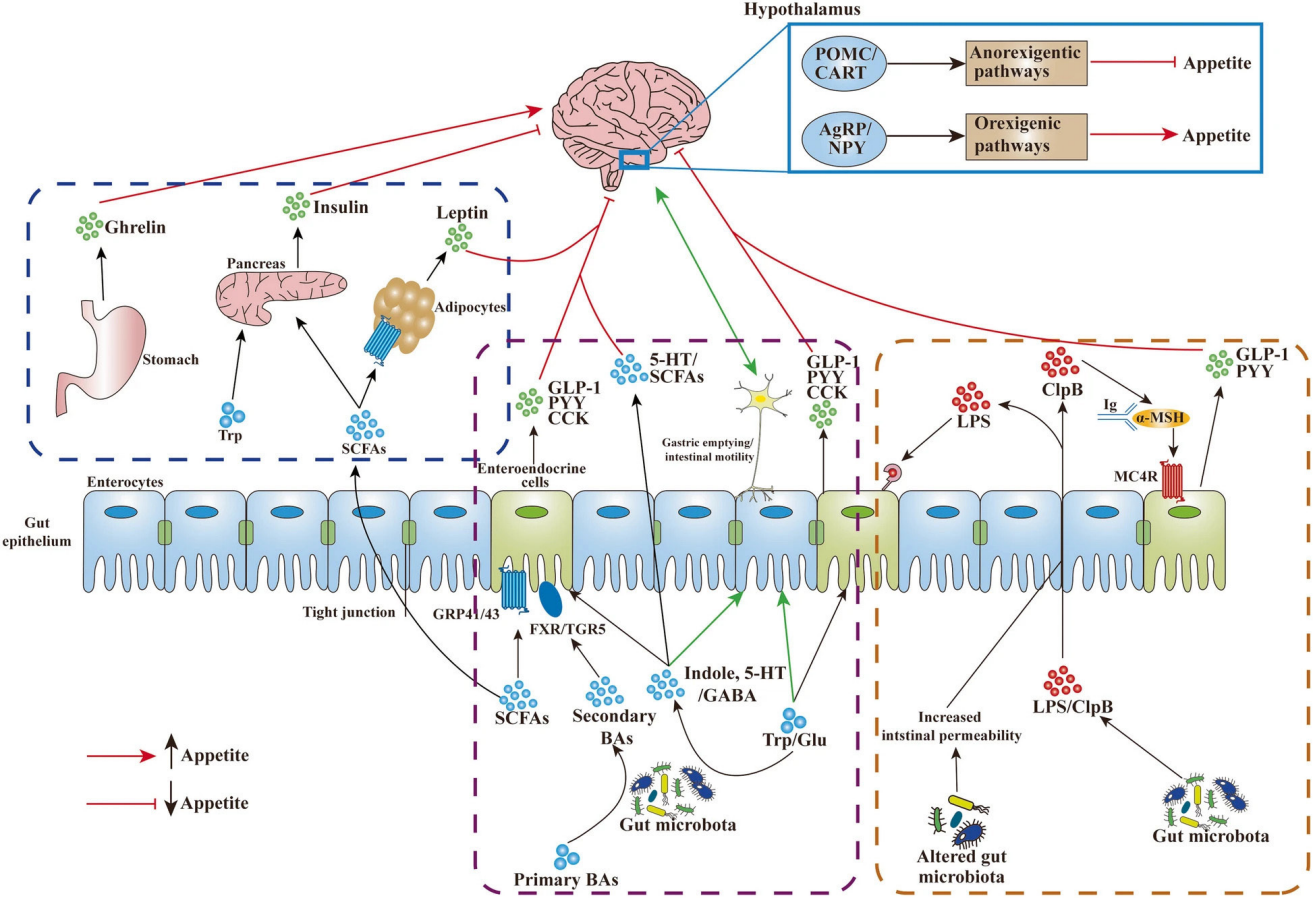
A complex network interconnects various components of the gastrointestinal tract, pancreas and adipocytes in the body with diverse products of the dynamic enteric microbiome, exerting influence on hunger, appetite, satiety and food-seeking behaviour in humans. Mechanisms associated with gut microbiota play a crucial role in regulating host appetite. Firstly, metabolites produced by gut microbes can stimulate enteroendocrine cells, leading to the release of anorexigenic hormones (PYY, GLP-1 and CCK) and neurotransmitters (5-HT), as well as the secretion of peripheral hormones (leptin, ghrelin and insulin). Secondly, immunoglobulins (Igs) play a role in modulating the biological activity of appetite-regulating hormones like leptin and ghrelin. Furthermore, the gut microbiota can generate proteins with sequences identical to those of appetite-regulating peptides, such as ClpB (caseinolytic protein B). These proteins may directly impact anorexigenic neurons or bind to Igs, thereby influencing the secretion of anorexigenic hormones from enteroendocrine L cells. CART, cocaine and amphetamine regulating transcript; POMC, proopiomelanocortin; LPS, lipopolysaccharide endotoxin. Reproduced from Figure 1 in [78]. CC BY (https://creativecommons.org/licenses/by/4.0/).

Enteric microbiome acts through the central nervous system through various metabolic products which either increase the leakiness of the blood-brain barrier increasing the likelihood of passage of many chemicals to affect neurotransmission [82] or they produce a series of neurotransmitters through tryptophan [83] or tyrosine metabolism or else they allow increased production of amino acids like glutamine that can pass into the brain to cause downstream action [84,85]. Products of tryptophan metabolism may also include vitamin B3 or nicotinamide. This has many functions in various intermediary metabolisms involving dehydrogenase reactions and in immunomodulation. Some of the metabolites of tryptophan and other aromatic amino acids are metabolized by the enteric microbiome and similar aromatic ring-containing material in the food works through aryl hydrocarbon (ArH) receptors which are present in enterocytes and many other cells to produce cytokines, angiogenesis-inducing molecules and xenobiotic transformation enzymes via increased transcription (Fig. 4). These cytokines can also directly alter the metabolic function in the brain.

Some other mechanism involving the brain works indirectly through the elaboration of adipocyte hormones from adipocyte secretome (Fig. 1) or hormones from the gastrointestinal tract which affects appetite and hunger [62,80] as already described. Yet another way the enteric microbiome can involve the central nervous system is through the increase of various cytokines either increased through the action of endotoxin of the bacteria or by indirect action of these endotoxins through many immune effector cells by acting as a pathogen-associated molecular pattern (PAMP). Appetite, hunger and satiety mechanisms in the brain intricately involve the enteric microbiome through a complex network (Figs4  5) of action, and its afferent/efferent pathways involve all our olfactory and taste receptors, vagus nerve and enteric neurons in addition to various hormones elaborated by this action [70].

**Fig. 5.**
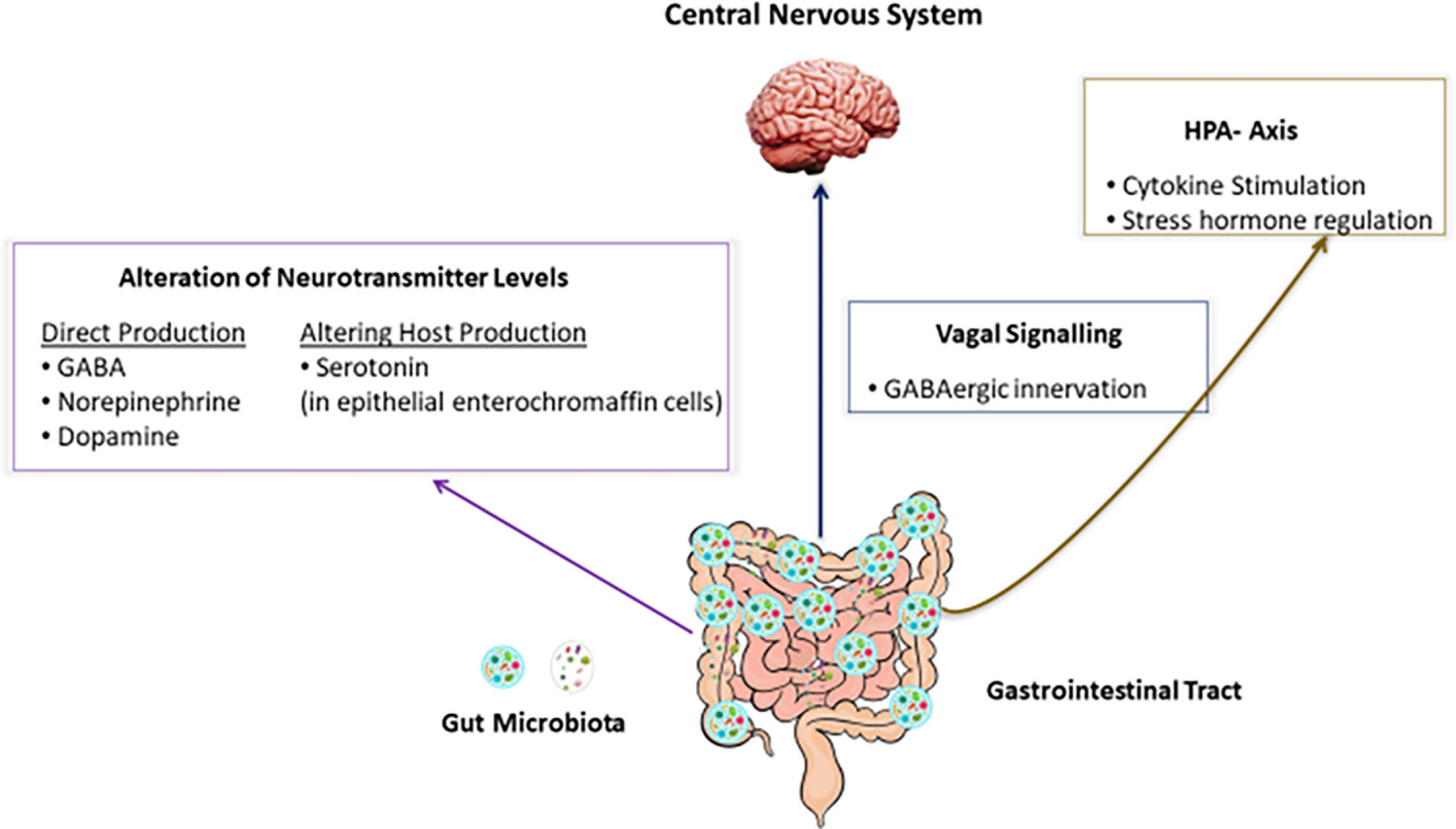
Communication routes of the gut microbiota to the brain. The gut microbiota has been found to communicate with the brain through several different mechanisms. This includes production of neurotransmitters or modulation of host neurotransmitter catabolism, innervation via the vagus nerve or activation of the hypothalamic-pituitary-adrenal axis. Adapted from [140].

### Production of targeting ligands by the enteric microbiome influences various local and systemic functions

Products from the enteric microbiome originate from the digestion of roughage material containing complex polysaccharides, mucopolysaccharides from secreted mucus, undigested proteins and lipids from the food. In addition to these dietary residues, the colon also receives bile acids, unabsorbed vitamins and residual digestive enzymes. A large number of intestinal cells that undergo apoptosis (average life span of intestinal epithelial cells is about 7 days) and products of bacterial metabolism as well as material which originates from dead and decomposing bacteria including endotoxin contribute to the diversity of such products.

Broadly, these products are SCFAs, i.e. acetic, propionic, butyric acids, hydroxy fatty acids and amino acids, di and tri-peptides, aryl hydrocarbon derivatives, complex products of bacterial metabolism, nucleotides, several vitamins and interestingly a couple of gases like methane (methanogenic bacteria), carbon dioxide and monoxide, hydrogen sulphide, ammonia, hydrogen and nitric oxide to name a few. Subsequently, many of these gases are utilized in oxidation-reduction reactions, conjugation, transamination and purine biosynthesis. Variability of the enteric microbiome and food composition brings immense diversity in the detailed composition of the products, but the broad classes of products produced have been described here.

Endotoxin from the bacterial cell wall is also an important effector molecule. These end products act locally on intestinal epithelial cells, resident and migrating immune cells and after absorption in distal cells and tissues targeting various receptors, some of which have already been described. Some of these products also affect directly the local as well as the distal nervous system (Fig. 5). These compounds engage various receptors like GPCR of various types, aryl hydrocarbon receptors, free fatty acid receptors, bile acid receptors (FXR, RXR, GPBR1 and S1PR2), dopamine, serotonin receptors and receptors for polyamines such as glutamine and GABA [84,89].

### Production of vitamins by the enteric microbiome

The enteric microbiome synthesizes many water-soluble vitamins like B1, B2, B3, folic acid, vitamin B12, biotin, pantothenic acid and coenzyme Q. In addition, it also synthesizes fat-soluble vitamin K [90,91]. Many of the vitamins supplied in the diet alter the composition and rate of growth of different members of the microbiome such that there is a two-way interaction between vitamins and gut microbiome [92]. For example, B12 supplementation has been shown to shift microbial community composition and SCFA output in a donor-dependent manner [93]. Vitamins themselves are involved in many biochemical reactions like dehydrogenation, decarboxylation, carboxylation and methyl transfer. Some of the vitamins also act as ligands for nuclear and other receptors influencing host transcriptional responses. Moreover, gut microbes not only synthesize but also compete for and transform vitamins. Many bacteria are auxotrophic and must scavenge vitamins like B12 using high-affinity transporters, which can limit availability to other microbes or the host [94]. Additionally, certain microbes enzymatically convert vitamins into more bioactive or bioavailable forms; for instance, vitamin K1 (phylloquinone) is converted into vitamin K2 (menaquinone) in the colon, enhancing its physiological impact [95]. Previously, it was believed that vitamins that are produced in the colonic environment are not absorbed, but current evidence shows this is not the case [96].

Though vitamin D is not directly produced by the microbiota, it alters the metabolism of internally produced or absorbed vitamin D from dietary sources, increasing 25 hydroxyvitaminD and 24,25 dihydroxyvitamin D along with improvement of calcium homeostasis. This function happens through the suppression of FGF23 production through the enteric microbiome [97]. A large number of vitamins obtained directly through food or microbiome help in the production and differentiation of various subsets of T cells or cause apoptosis of certain other cells by complex molecular interaction which has been well described elsewhere [98,99].

### Immunomodulation and creation of cytokine milieu of chronic inflammation

The intestinal tract especially the colonic mucosa is exposed to a huge number of bacterial products either through the transformation of food residues outside the bacterial body or as products of intermediary metabolism of bacterial cells which by the action of a community of different bacteria can interact with one after another as a chain on a molecule to transform it. Finally, the dead or decaying bacteria produce bacterial endotoxin which stimulates chronic inflammation by TLR 4 engagement working on intestinal epithelial cells as well as after absorption on hepatic, adipose and many other immune competent cells, creating a state of chronic inflammation that positively contributes to the pathologic condition. The intestinal immune system plays a pivotal role in shaping microbial communities, especially through secretory immunoglobulin A (sIgA) and dendritic cells. sIgA binds to microbial antigens, promoting mucosal tolerance and spatial compartmentalization of commensals [100]. Dendritic cells, on the other hand, continuously sample luminal contents via trans-epithelial dendrites or M-cell transcytosis and present these antigens to the gut-associated lymphoid tissue, influencing T cell differentiation and maintaining immune tolerance [101]. These immune–microbiota dynamics are critical for maintaining barrier integrity and metabolic balance, and disruptions can exacerbate chronic low-grade inflammation characteristic of obesity. Foods that induce a chronic inflammatory state such as highly refined carbohydrates and high-fat meals, defined as diets where more than 30% of caloric intake comes from fats and deep-fried foods which introduce oxidized lipids and other harmful compounds due to high-temperature processing, induce a chronic inflammatory state contributing to the pathologic condition [99,102,106].

**Fig. 6.**
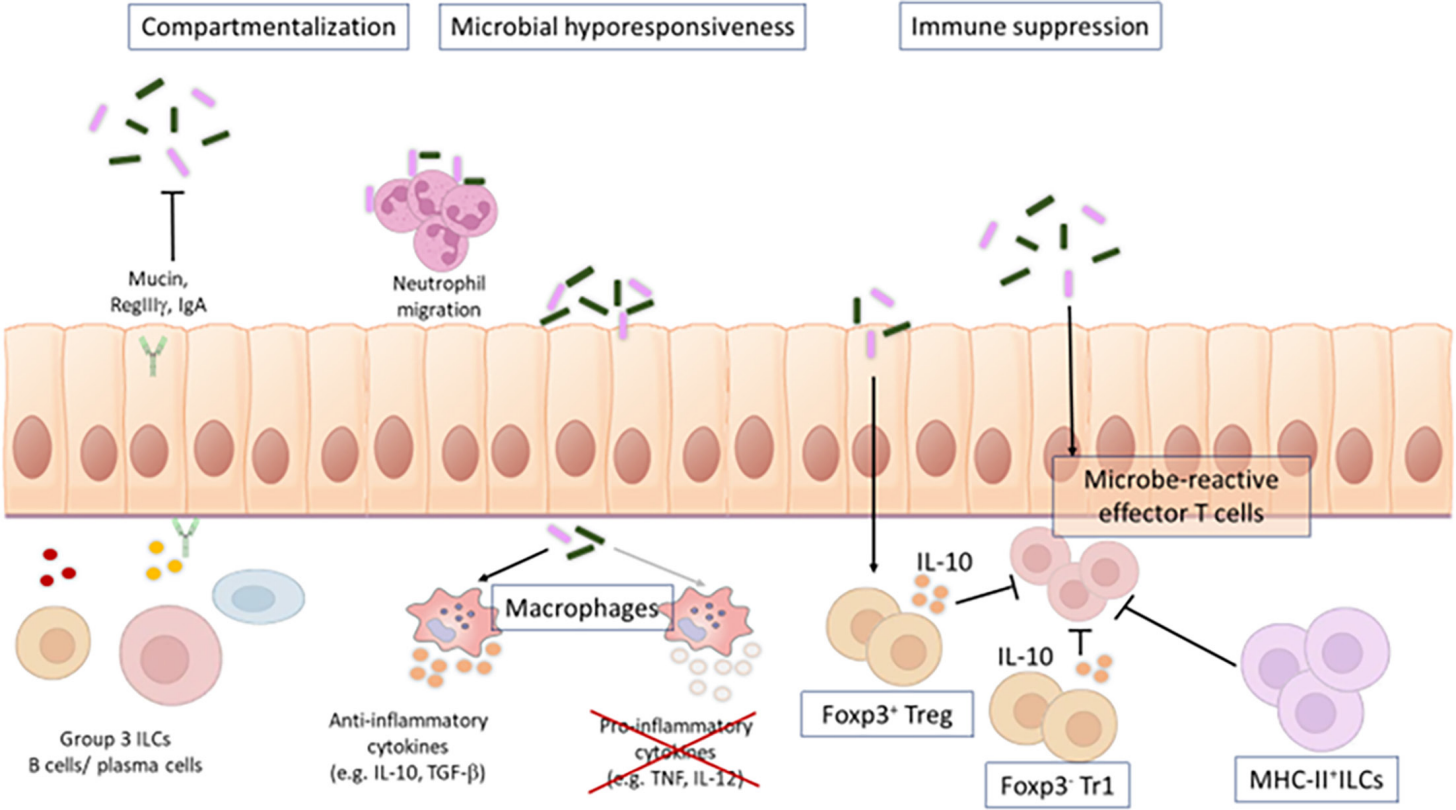
Multiple firewalls limit immune responses against resident intestinal bacteria. The intestine has firewalls that limit inappropriate immune responses against commensal bacteria. One mechanism involves compartmentalization of the resident bacteria by a thick mucus layer, anti-microbial peptides or secreted IgA, which all prevent access of the resident bacteria to the epithelium. Neutrophils can migrate out to the intestinal lumen and build intraluminal cellular casts that encapsulate commensals, limiting the penetration of luminal microbes. A second mechanism involves microbial hyporesponsiveness of resident intestinal macrophages. In contrast to monocytes and macrophages in systemic tissues, intestinal macrophages are hypo-responsive to microbial stimuli and produce only limited amounts of inflammatory cytokines such as tumour necrosis factor-alpha or IL-12. Furthermore, resident phagocytes spontaneously produce anti-inflammatory cytokines, such as IL-10. A third mechanism involves active suppression of microbe-reactive effector T cell responses by Foxp3+iTreg cells and Foxp3− Tr1 cells via production of IL-10. In addition, MHC-II+ILCs present bacterial antigens, which limit the activation of commensal-reactive CD4+T cells. Adapted from [103].

Entry and interaction of endotoxin or bacteria themselves and some of the PAMPs produced by the microbiome are variably blocked access or entry by a layer of mucus that sticks to the epithelial cell layer of the intestine. The mucus-degrading capability of some of the microbes in the microbiome by digesting the barrier mucus variably contributes to chronic inflammation [107,109]. Immunomodulation by enteric microbiome inside the intestine has been well depicted in Fig. 6.

### Epigenetic regulation of the host genome

Various products of the enteric microbiome as already described not only modify the host transcriptome and metabolome through its various products directly or through various indirect modes by acting as ligands for the network of messengers systems in immune and non-immune cells but also through modulation via central, autonomic and enteric neuronal pathways or via various true/false neurotransmitters like octopamine, tyramine and derivatives of dopamine pathways using products obtained from food/drugs following the microbiome’s modification thereof.

Epigenetic changes induced by the microbial products of the enteric transcriptome are yet another way of altering the acquired transcriptomic landscape of the host, giving the active genome of the host an individuality that is an act of nature as well as that of nurture. It is increasingly being recognized that epigenetic regulation is a potent mechanism for the enteric microbiome to influence host physiology. This can occur via many potential mechanisms: (1) activation of host-cell intrinsic processes directing epigenetic modulation, (2) regulating expression or/and activity of epigenetic modifying enzymes, (3) microbial biosynthesis or metabolism which influence the availability of donor radicals or chemical modifiers of host DNA or histone [110,111]. This is achieved by DNA/histone methyltransferases (DNMTs/HMTs) and histone acetyltransferases (HATs). These enzymes rely on methyl and acetyl donors, respectively, for their catalytic activity [111]. The enteric microbiome synthesizes a large number of SCFAs or hydroxy fatty acids and in addition through its methyl donor metabolism either via folate/B12 pathway (a part contributed by the microbiome) or through methanogenic bacteria. Bile acid metabolites also change the signature through GPCR receptor engagement [104]. In addition to SCFAs and bile acid derivatives, gut microbes produce amine metabolites with systemic effects. Tyramine, produced by microbial tyrosine decarboxylases, functions as a trace amine affecting neurotransmission and vascular tone via catecholamine release and trace amine-associated receptor (TAAR1) activation [112]. Phenylacetic acid, derived from microbial phenylalanine metabolism (notably via *Bacteroides*), promotes hepatic triglyceride accumulation and attenuates insulin-stimulated AKT (protein kinase B) activation, implicating it in lipogenesis and insulin resistance [113]. Polyamines are one of the important products originating from the interaction of food and microbiome. Polyamines such as putrescine and spermidine are derived from dietary amino acids like arginine and ornithine and are synthesized by both microbes and the host, often via cross-fed pathways. These products regulate epithelial cell proliferation and are important genetic and epigenetic regulators of gene function in the host [114,116].

### Microbiota-mediated modulation of host energy expenditure and thermogenesis

In terms of energy balance, this pathologic condition is about positive energy balance. This accrues from dietary input of absorbable food, extra absorbable food material that is created by both energy metabolism and digestion of roughage as well as mucus and microbiota-produced products through their intermediary metabolism. The enteric microbiome may increase gut permeability, influencing the central nervous system through the absorption of food and ultimately altering food-seeking behaviour via changes in appetite produced by the microbiome, as previously described.

This calorie is burned by thermogenesis, temperature regulatory mechanisms, work and working conditions and stimulation or suppression of metabolism through neuroendocrine and mitochondrial mechanisms (uncoupling or coupling of oxidative phosphorylation). This process has been studied and reviewed in detail elsewhere [15,40, 48, 49, 117,120]. Energy expenditure may be increased or decreased through mitochondrial metabolism [76] or by inducing endoplasmic stress [120] with a suitable combination of input diet and microbiota.

### Bariatric surgery changes gut microbiome

Bariatric surgery is now regularly used for this morbid pathologic condition and other types of pathologic conditions like type 2 diabetes where there are comorbid conditions and weight-reducing therapy failed. However, this surgery is not used below a BMI of 30. It was believed for a long time that a reduction in food intake is due to the reduction of stomach capacity or the reduction of the area of absorption bypassing the variable length of the small intestine (Roux-en-Y gastric bypass procedure). There are several varieties of bariatric procedures [121] that are suitable for different degrees of this pathologic condition. Nowadays, one of the main reasons for weight loss following this surgery is believed to be an alteration of enteroendocrine secretion due to the procedure itself and/or due to changes in the enteric microbiome [121,123]. This change in the composition of the microbiome may be responsible for the persistent effect on this condition. The effect of this type of surgery lasts even when over time the food intake improves [121]. Different types of bariatric surgery were shown to influence the enteric microbiome differently, thereby partly influencing the variation in results obtained from different types of bariatric surgery [124].

### Antioxidant chemicals from the microbiome

It has been already described that chronic inflammation is an important companion of this pathologic condition. The immune system, adipose tissue and enteric microbiome are co-conspirators for continuing the chronic inflammatory state associated with the pathologic condition [24,72, 84]. The chronic inflammatory state is often continued through pro-oxidant chemicals in food. Dietary polyphenols are important antioxidants. However, many of them are not absorbed from the small intestine because the antioxidant moiety of polyphenols often exists as complex organic compounds. These compounds can be broken down into absorbable simple polyphenols by some of the members of the microbiome [103,125,128] and assist in increasing net antioxidant input from food and food residues.

### The pathologic condition-inducing microbiome exists, and it is possible to manipulate it

Several studies investigating the enteric microbiome in individuals with obesity [48,128], including those with polycystic ovarian syndrome, a condition frequently associated with obesity [129], have demonstrated distinct alterations in gut microbial composition. While inter-individual variation exists, a consistent signature across many patients is characterized by an increased relative abundance of Firmicutes and a decreased proportion of *Bacteroides*, forming what is often referred to as a core obesity-associated microbiome [48,61, 128]. Studies on twins discordant for obesity also showed similar changes, and studies on uniovular and other twins discordant for obesity clearly showed that the impact and composition of the enteric microbiome associated with the pathologic condition is more of an environmental contribution rather than the host’s genetic makeup.

Studies involving twins discordant for obesity, analyses of the enteric microbiome in individuals with the obese phenotype and comparisons across geographically distinct populations with varying obesity prevalence, as well as FMT and dietary intervention studies including post-bariatric surgery microbiome shifts, all suggest that the gut microbiome can be modulated through multiple strategies to help manage obesity. These approaches complement existing methods targeting appetite and food-reward pathways via pharmacologic and behavioural interventions. However, as already referred to, appetite and satiety behaviour can also be controlled by many chemicals from the interaction of the microbiome with food residues and the products of its own metabolism [62,70, 80, 82,85] (Figs1, 2, 3, 6  7). The pathology-induced microbiome also alters food preference and food-seeking behaviour [130,133].

**Fig. 7.**
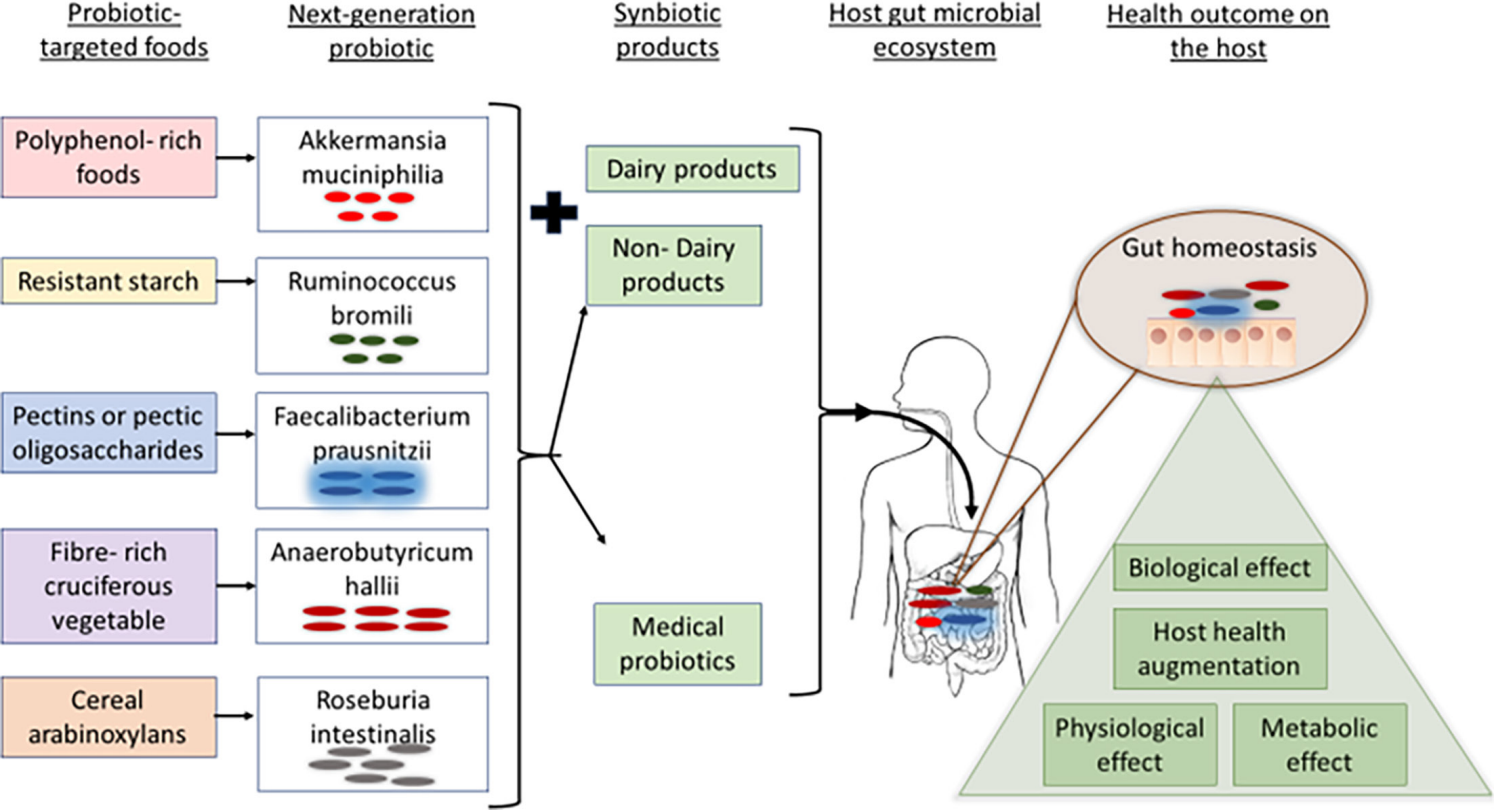
Approach to modify the enteric microbiome for biological effects, including treatment of obesity. Specific probiotic-targeted foods and next-generation probiotics can be combined as synbiotic products to influence the host gut microbial ecosystem, supporting gut homeostasis and delivering biological, physiological and metabolic benefits. Adapted from [137].

### Engineering therapeutic gut bacteria and development of probiotic food programme to fight the pathologic condition

The present review so far described how the enteric microbiome, via many of its interactions with food residues and the host, creates a situation that is conducive to obesity and leanness. Cross-faecal transplantation experiments have demonstrated that susceptibility to leanness or obesity can be transferred between animals, with microbiota from lean donors promoting a lean phenotype in obese recipients and vice versa. Moreover, in patients with obesity, there is a general tendency to increase in *Firmicutes* at the expense of *Prevotella* and *Bacteroides* in its core microbiome. Similar changes are seen with ageing and with western types of high meat, high fat, refined carbohydrate and low roughage food. Hence, it should be possible to create probiotic/prebiotic food supplements or design natural food composition to fight the pathologic condition along with other means such as drugs and calorie restriction coupled with exercise. Drugs targeting gut hormone receptors are becoming very effective tools in fighting the condition [5,10]. In the same way, probiotic food/next generation of probiotic and symbiotic or bioengineered bacteria or its products can be suitably used for individualized management of the condition (Fig. 7) [134,137]. The use of biotechnology to identify, select and engineer beneficial bacterial strains or their metabolites has been envisioned as a multi-step process [136].

## Discussion and conclusion

Oral, cutaneous, genito-urinary and enteric microbiomes altogether constitute an extra organismal metabolic organ that in various ways influence and interact with the genetics and biology of the host. That is why even when two individuals have the same genetic makeup and apparently stay in a similar environment, they may have variable long- and short-term effects due to the imposition of this microbiomic organ function on the host.

Of all the five areas of microbiome in the body, enteric microbiome is the most extensive and dynamic. Though this microbiome constitutes a large number of bacterial species from different genera and species, almost 70% of them are non-culturable and their function is inferred from their genetic signature using large-scale 16s molecular ribotyping and whole-genome sequencing along with modern software-based computation called metagenomic studies [21,22, 25, 26]. This area of exploration despite the large-scale data acquisition is incomplete. Recent advances in culturomics [33,37] are contributing to a better understanding of complementary biochemical and molecular interactions of various bacterial species. The web of interaction of enteric microbiome through their complementary biochemical reaction is capable of processing various roughage containing polysaccharides, undigested proteins, mucins, apoptotic intestinal epithelial cells by producing hundreds of chemical entities like SCFAs, hydroxy fatty acids, various peptides, amino acids, products of amino acid catabolism having neurotransmitter function and antigenic moieties through the bacterial cell wall, endotoxins and other chemicals influence the immune system, trigger the secretion of incretins and other gut hormones, influence the intestinal nervous system and central nervous system directly or indirectly. Through stimulation of various cells and influencing epigenetic changes by DNA methylation and histone modification and by changing the permeability of gut epithelium, these products influence appetite, hunger, food storage, food seeking and food eating behaviour, food preference and energy balance in the body. By coordinating the intestinal nervous system with the activity of the vagus nerve and central neuronal outflow in addition to hormonal control, the microbiome also controls the gastric emptying time, intestinal transit time and peristalsis.

Absorbed endotoxins and fragments of DNA engage toll-like receptors in intestinal cells and in immunocompetent cells of the intestine, as do various vitamins synthesized by the enteric microbiome. These stimulations or ligand receptor interactions produce a dynamic cytokine network, which continues to influence many areas of biology that lead to the pathologic condition.

Metabolism of bile acids and other aromatic compounds in food produces many derivatives that can activate several GPCR receptors in the intestine as well as in various cells of the body. Aryl hydrocarbon produced can engage various types of aryl hydrocarbon receptors producing several important biological actions.

It is also seen that though there is innumerable variation in details of the enteric microbiome in different periods of life with lean and the pathologic phenotypes, there is a discernible core microbiome with *Firmicutes*, *Bacteroides*, *Prevotella*, *Ruminococcus* and *Lactobacillus*. Pathologic and lean phenotype individuals have broadly different microbiomes in a quantitative manner with *Bacteroides* and *Prevotella* predominating in lean phenotype.

Faecal transplants in animals can convert a lean type of animal to pathologic phenotype and vice versa using proper stool samples. All these findings demonstrate that it will be possible to change the enteric microbiome by using probiotics with select bacterial species such as *Lactobacillus* which is already available and using improved prebiotics and probiotics and improved bacteria by using recombinant DNA and other technologies [133,137].

Our understanding of the enteric microbiome is still very incomplete. Apart from bacterial species, the microbiome also consists of different types of yeasts, candida, various types of viruses and phages to complete the list. Very little is known about the contribution of this vast majority of yet unaccounted microbes.

One of the very important findings about the enteric microbiome is that at the time of delivery, this microbiome already becomes discernibly different in babies born naturally and those born following caesarean section. For the first 3 years of life, this microbiome which is simple initially becomes complex as different types of food are introduced into the baby’s diet and breast milk.

In the initial period of infancy and childhood repeated exposure to antibiotics changes the microbiome to a future pathologic phenotype, and this could be an important component of obesity starting in childhood [138], and that will be the ideal time for preventive management through long-term dietary/microbiomic manipulation.

## Abbreviations

FMT: faecal microbiota transplantation
PAMP: pathogen-associated molecular pattern
SCFA: short-chain fatty acid
sIgA: secretory immunoglobulin A
